# Eating disorders among patients with borderline personality disorder: understanding the prevalence and psychopathology

**DOI:** 10.1186/s40337-020-00314-3

**Published:** 2020-08-17

**Authors:** Mohsen Khosravi

**Affiliations:** Department of Psychiatry and Clinical Psychology, Baharan Psychiatric Hospital, Zahedan University of Medical Sciences, Zahedan, 9813913777 Iran

**Keywords:** Alexithymia, Anxiety, Borderline personality disorder, Depression, Feeding and eating disorders, Psychopathology

## Abstract

**Background:**

Treatment protocols can be bolstered and etiological and maintenance factors can be recognized more easily by a superior understanding of emotions and emotion regulation in the comorbidity of borderline personality disorder (BPD) and feeding and eating disorders (FEDs). Therefore, the present study aimed at investigating the prevalence and psychopathology of FEDs in patients with BPD.

**Methods:**

In this cross-sectional study, 220 participants were examined in three groups, namely BPD (*n* = 38), BPD + FEDs (*n* = 72), and healthy controls (*n* = 110), from August 2018 to November 2019. The participants were selected by systematic random sampling among the patients who referred to Baharan psychiatric hospital in Zahedan, Iran, with the sampling interval of 3. The subjects were evaluated by 28-item General Health Questionnaire (GHQ-28), Borderline Personality Inventory (BPI), Structured Clinical Interview for DSM-5 Personality Disorders (SCID-5-PD), Structured Clinical Interviews for DSM-5: Research Version (SCID-5-RV), the 26-item Eating Attitudes Test (EAT-26), 20-item Toronto Alexithymia Scale (TAS-20), Beck Anxiety Inventory (BAI), and Beck Depression Inventory-II (BDI-II).

**Results:**

The results showed a 65.4% (*n* = 72) prevalence of FEDs in patients with BPD. Also, the highest and lowest prevalence rates were reported for other specified feeding and eating disorders (51.3%) and bulimia nervosa (6.9%), respectively. Although the highest mean score of TAS-20 was related to anorexia nervosa, there was no significant difference between the scores of various types of FEDs. The mediation analysis showed that anxiety and depression would play a mediating role in the relationship between alexithymia and eating-disordered behaviors.

**Conclusions:**

The results have suggested that alexithymia, anxiety, and depression should receive clinical attention as potential therapeutic targets in the comorbidity of BPD and FEDs. The clinical implications of the research have been conducted to date, and directions for future research have been discussed.

## Plain English summary

Feeding and eating disorders (FEDs) and borderline personality disorder (BPD) are relatively common psychiatric problems amongst populations. Many people suffering from these disorders also have difficulties dealing with their emotions: they struggle to recognize and talk about their emotions (a psychological characteristic called “alexithymia”) and regulate their emotions appropriately. Common comorbidity and shared psychopathology in BPD and FEDs conceptualize the relationship between eating-disordered behaviors, alexithymia, depression, and anxiety. An improved understanding of the role of emotions in the comorbidity of BPD and FEDs can help screening, enhance treatment protocols, and provide a better understanding of the etiological and maintenance factors involved in this comorbidity. Accordingly, it seems necessary to investigate the relationship between alexithymia, anxiety, depression, and eating-disordered behaviors in more detail. Therefore, the present study aimed at investigating the prevalence and psychopathology of FEDs in patients with BPD.

## Background

Borderline personality disorder (BPD) is a chronic and disabling disorder, imposing many costs on societies through severe functional impairments, high risk of suicide, negative effect on the course of depressive disorders, and extensive use of treatments [1]. Clinical experience suggests that disordered-eating behavior and actual feeding and eating disorders (FEDs) are relatively common among patients with BPD such that the rate of this comorbidity has been reported at 14–53% in different studies [2–4]. Thus, this question is raised: “Is this high comorbidity an indicator of a partially causal relationship?” [5].

Despite expanded studies, the question is still unanswered [2]. In 2001, Dolan et al. [6] proposed a useful framework for developing different theoretical models to investigate the comorbidity of psychiatric disorders. For instance, the spectrum/subclinical model supposed that BPD and FEDs are similar concerning etiologies and action mechanisms [2, 6]. Based on this assumption, it can be inferred that the use of emotion regulation strategies, including rumination, suppression, and avoidance by BPD and FEDs patients, may highlight the important role of emotion regulation (as a transdiagnostic construct) in the evolution of these disorders [7].

In this respect, researchers have indicated that alexithymia (i.e., inability to recognize and express the emotions) can be involved in many types of psychopathologies, such as BPD and FEDs, by preventing the regulation of negative emotions [8, 9]. For example, Zlotnick et al. [10] and Wolff et al. [11] indicated that patients with BPD could not usually recognize emotions and their causes. Moreover, Nowakowski et al. [12] demonstrated that individuals with FEDs consistently reported higher levels of alexithymia on the 20-item Toronto Alexithymia Scale (TAS-20) compared to healthy controls. Accordingly, they suggested that patients used eating-disordered behaviors to control their emotional state.

Although there is evidence for the alexithymia effect on emotion regulation, little information has been obtained about this process so far [13]. Nevertheless, recent studies have suggested that unawareness of emotions results in the inability to regulate emotions successfully and, in turn, autonomic arousal is chronically increased [14]. Pandey and Mandal [15] suggested that the relationship between alexithymia and overestimated perceived arousal is due to the association of alexithymia with anxiety. Later, following the introduction of these issues, several authors considered alexithymia a personality trait that can give rise to anxiety and depression by causing problems in the management of emotions, anxiety, and depression [16]. Furthermore, the results of several studies suggested that, compared with the normal population, both anxiety and depression are significantly more prevalent among patients with comorbidity of BPD and FEDs, which can be mainly attributed to high levels of alexithymia in these patients [12, 13, 16]. In this regard, alexithymia could enhance anxiety and depression due to low self-esteem and insecurity. These secondary emotions increase the patients’ distress and reduce their coping abilities, thereby leading to engagement in eating-disordered behaviors (e.g., bingeing, purging, and dietary restriction) in an effort to avoid or cope with the emotions [12, 16, 17].

Thus, given that FEDs is a subtype of emotional disorders, deficits in emotional processing observed in FEDs should be fully mediated by anxiety and depression. In other words, if FEDs do not constitute a distinct pathology from emotional disturbances, only anxiety and depression must mediate the relationship between alexithymia and eating-disordered behaviors [18, 19]. Consistent with the complete mediation hypothesis, Eizaguirre et al. [16] revealed that anxiety and depression can mediate the relationship between alexithymia and eating-disordered behaviors.

Moreover, an improved understanding of the role of emotions in the comorbidity of BPD and FEDs can help screening, enhance treatment protocols, and provide a better understanding of the involved etiological and maintenance factors. Therefore, it seems necessary to investigate the relationship between alexithymia, anxiety, depression, and eating-disordered behaviors in further detail. For this purpose, the present research pursues three goals, including (1) comparing the mean scores of Beck Anxiety Inventory (BAI), Beck Depression Inventory-II (BDI-II), TAS-20, the 26-item Eating Attitudes Test (EAT-26), and 28-item General Health Questionnaire (GHQ-28) for the three study groups (including BPD, BPD + FEDs, and healthy controls); (2) comparing the mean scores of TAS-20 in terms of different FEDs subtypes in BPD + FEDs patients; (3) investigating the mediating role of anxiety and depression in the relationship between alexithymia and eating-disordered behaviors in both BPD only and BPD + FEDs groups.

## Methods

### Participants

This cross-sectional study was performed from August 2018 to November 2019. In this study, 110 patients with BPD and 110 healthy people were selected. The sample size was calculated based on the study performed by Eizaguirre et al. [16]. Power calculations for 80% power to detect and effect size of r = 0.3 at alpha = 0.5 yielded a necessary sample of *N* = 220 [20]. The patients with BPD were selected by systematic random sampling among the persons who referred to Baharan psychiatric hospital in Zahedan, Iran, with the sampling interval of 3. Also, the healthy people were selected out of their relatives through one-to-n matching (i.e., one case dedicated to one control). The inclusion criteria were as follows: (1) getting a score above 10 in Borderline Personality Inventory (BPI) and approved diagnosis of the disorder based on Structured Clinical Interview for DSM-5 Personality Disorders (SCID-5-PD) by a psychiatrist; (2) age range of 18–35 years; (3) ability to read and write alongside reading comprehension; (4) for healthy people, getting a score of ≤22 in GHQ-28 and approved mental health based on Structured Clinical Interviews for DSM-5: Research Version (SCID-5-RV) and SCID-5-PD by the psychiatrist. The exclusion criteria included the followings: (1) severe and acute physical illness; (2) brain traumatic injury; (3) comorbidity of schizophrenia and other psychotic disorders; (4) epileptic disorder; (5) intellectual disability; (6) mixed personality disorder; (7) taking any drug or substance that causes anorexia and bulimia; (8) failing to fill the questionnaires properly.

In this study, 220 participants were evaluated in three groups, namely BPD (*n* = 38), BPD + FEDs (*n* = 72), and healthy controls (*n* = 110). Table 1 presents the participants’ sociodemographic information. According to the table, there was no significant difference in sociodemographic factors between the study groups.

**Table 1:** Comparisons of the socio-demographic factors, BAI, BDI-II, TAS-20, EAT-26, and GHQ-28 among the three study groups (*N* = 220)

Variables	Subvariables	Healthy Controls (*n* = 110)	BPD (*n* = 38)	BPD + FEDs (*n* = 72)		
		*n* (%)	*n* (%)	*n* (%)	*χ*^2^ (2)	
		37 (33.6)	10 (26.3)	23 (31.9)	0.71	
Age	18–23					
	24–29	43 (39.1)	16 (42.1)	26 (36.1)		
	30–35	30 (27.3)	12 (31.6)	23 (31.9)		
Gender	Male	28 (25.5)	17 (44.7)	26 (36.1)	5.26	
	Female	82 (74.5)	21 (55.3)	46 (63.9)		
Marital status	Never married	57 (51.8)	22 (57.9)	34 (47.2)	17.71	
	Married	28 (25.5)	16 (42.1)	18 (25.0)		
	Cohabiting	5 (4.5)	0 (0)	2 (2.8)		
	Widowed	2 (1.8)	0 (0)	5 (6.9)		
	Divorced	13 (11.8)	0 (0)	8 (11.1)		
	Separated	5 (4.5)	0 (0)	5 (6.9)		
Education level	Non-degree	45 (40.9)	9 (23.7)	28 (38.9)	1.84	
	High school diploma	38 (34.5)	4 (10.5)	23 (31.9)		
	Academic degree	27 (24.5)	25 (65.8)	21 (29.2)		
Income	< 10,000,000 Rials	73 (66.4)	23 (60.5)	44 (61.1)	0.70	
	≥ 10,000,000 Rials	37 (33.6)	15 (39.5)	28 (38.9)		
		M (SD)	M (SD)	M (SD)	F (2, 217)	Scheffé post-hoc test
BAI		7.94 (6.20)	50.65 (8.91)	62.30 (2.86)	2005.08^***^	BPD + FEDs > BPD > Healthy Controls
BDI-II		3.53 (5.62)	26.07 (4.46)	38.90 (6.40)	866.85^***^	BPD + FEDs > BPD > Healthy Controls
TAS-20		28.10 (7.18)	53.94 (12.95)	79.00 (9.60)	671.54^***^	BPD + FEDs > BPD > Healthy Controls
EAT-26		1.32 (2.11)	8.55 (5.19)	28.08 (8.45)	521.03^***^	BPD + FEDs > BPD > Healthy Controls
GHQ-28		10.10 (5.48)	36.73 (7.09)	58.70 (11.30)	797.78^***^	BPD + FEDs > BPD > Healthy Controls

***Note.****BAI* Beck Anxiety Inventory, *BDI-II* Beck Depression Inventory-II, *BPD* Borderline personality disorder, *EAT-26* 26-item Eating Attitudes Test, *FEDs* Feeding and eating disorders, *TAS-20* 20-item Toronto Alexithymia Scale.

^*^p < 0.05; ^**^p < 0.01; ^***^p < 0.001.

### Procedures

After the approval of the research project by the ethics committee of the Medical Faculty of the Zahedan University of Medical Sciences (IR.ZAUMS.REC.1398.212), the informed consent forms were distributed among the participants. In order to follow the Declaration of Helsinki, participation in the study was optional, and the participants could leave the study for any reason. After obtaining informed consent from the participants, GHQ-28, EAT-26, TAS-20, BAI, and BDI-II were distributed among them. Next, the psychiatrist evaluated all of the participants using SCID-5-PD and SCID-5-RV to identify the three study groups (including BPD, BPD + FEDs, and healthy controls). The questionnaires were anonymous to keep the participants’ information private.

### Measures

The following measures were used in this study (in general, the Cronbach’s alpha values of 0.70 or higher indicate acceptable internal consistency) [21]:

#### TAS-20

Alexithymia was assessed with the Persian version of the TAS-20, a 20-item self-report questionnaire scored based on a five-point (1–5) Likert scale. The minimum and maximum scores are 20 and 100, respectively, and scores of ≥61 represent alexithymia [22]. In this study, the Cronbach’s alpha coefficients for the TAS-20 subscales of difficulty identifying feelings, difficulty describing feelings, and externally oriented thinking were 0.85, 0.82, and 0.75, respectively, while it was 0.72 for the total scale.

#### BAI

Anxiety symptoms were assessed with the Persian version of the BAI, a self-report 21-item questionnaire scored based on a four-point (0–3) Likert scale. The minimum and maximum scores are 0 and 63, respectively [23]. In this study, the Cronbach’s alpha coefficient for the BAI was 0.90.

#### BDI-II

Depressive symptoms were assessed with the Persian version of the BDI-II, a self-report 21-item questionnaire scored based on a four-point (0–3) Likert scale. The minimum and maximum scores are 0 and 63, respectively [24]. In this study, the Cronbach’s alpha coefficient for the BDI-II was 0.88.

#### EAT-26

Eating-disordered behaviors were assessed with the Persian version of the EAT-26. In this 26-item questionnaire, the minimum and maximum scores are equal to 0 and 78, respectively. A score above 20 stands for the probability of being affected by FEDs [25]. In this study, the Cronbach’s alpha coefficients for the EAT-26 subscales were as follows: drive for thinness = 0.90, restrained eating = 0.77, perceived social pressure to eat = 0.87, food preoccupation and oral control = 0.75 and bulimia = 0.71.

#### BPI

In this 53-item questionnaire (answered by yes or no), if the person’s score for the 20 items of the cutoff score is above 10, the person is most likely to be affected by BPD [26]. In this study, the Cronbach’s alpha coefficients for the BPI subscales of identity diffusion, primitive defense mechanisms, reality testing, and fear of closeness were 0.78, 0.82, 0.74, and 0.77, respectively, while it was 0.80 for the total scale.

#### SCID-5-PD

SCID-5-PD is a semi-structured clinical interview used by researchers and clinicians, which evaluates DSM-5 personality disorders under three clusters of A, B, and C, and other specific personality disorders. Several studies have reported acceptable reliability and validity of SCID-5-PD [27].

#### SCID-5-RV

SCID-5-RV is a semi-structured interview for major DSM-5 diagnoses, which is performed by a trained clinician or health expert familiar with the diagnostic criteria and classification of disorders in DSM-5. Several studies have reported acceptable reliability and validity of SCID-5-RV [28].

#### GHQ-28

GHQ-28 is a 28-item questionnaire in which items are scored in the range of 0–3. The overall score ranges between 0 and 84. A score of ≤22 indicates a person’s mental health [29]. In this study, the Cronbach’s alpha coefficients for the GHQ-28 subscales of somatic symptoms, anxiety and insomnia, social dysfunction, and severe depression were 0.76, 0.84, 0.71, and 0.88, respectively, while it was 0.90 for the total scale.

### Data analysis

Statistical analysis was performed using descriptive statistics, including mean and standard deviation. Chi square test and Kruskal-Wallis test were conducted for a sociodemographic comparison of the three study groups. Also, an analysis of variance (ANOVA) was used to compare the mean scores of BAI, BDI-II, TAS-20, EAT-26, and GHQ-28. In ANOVA, the Scheffé test was applied to the post-hoc analysis. Subsequently, in BPD + FEDs group, the Pearson correlation coefficient was used to evaluate the correlation between the study variables. Also, in this group, the mediation analysis was conducted to investigate the mediating effect of anxiety and depression on the relationship between alexithymia and eating-disordered behaviors using the Hayes’ PROCESS macro for SPSS [30]. As outlined in Preacher and Hayes [31], mediation emerges when the indirect effect is significant and the confidence intervals do not contain zero. Furthermore, given the relationship of sociodemographic factors (including age, gender, marital status, education level, and income) with anxiety, depression [32–35], and FEDs [36, 37] obtained in previous studies, the above-mentioned factors were considered as covariates in the mediation analysis. Meanwhile, data analysis was performed by SPSS 25, and the significance level was set at *P* < 0.05.

## Results

### Preliminary analysis

According to Table 1, there was a significant difference between the scores of BAI, BDI-II, TAS-20, EAT-26, and GHQ-28 among the three study groups. Based on Scheffé post-hoc test, the highest scores of BAI, BDI-II, TAS-20, EAT-26, and GHQ-28 were reported for BPD + EFD group, and the lowest scores were reported for healthy controls.

**Fig. 1:**
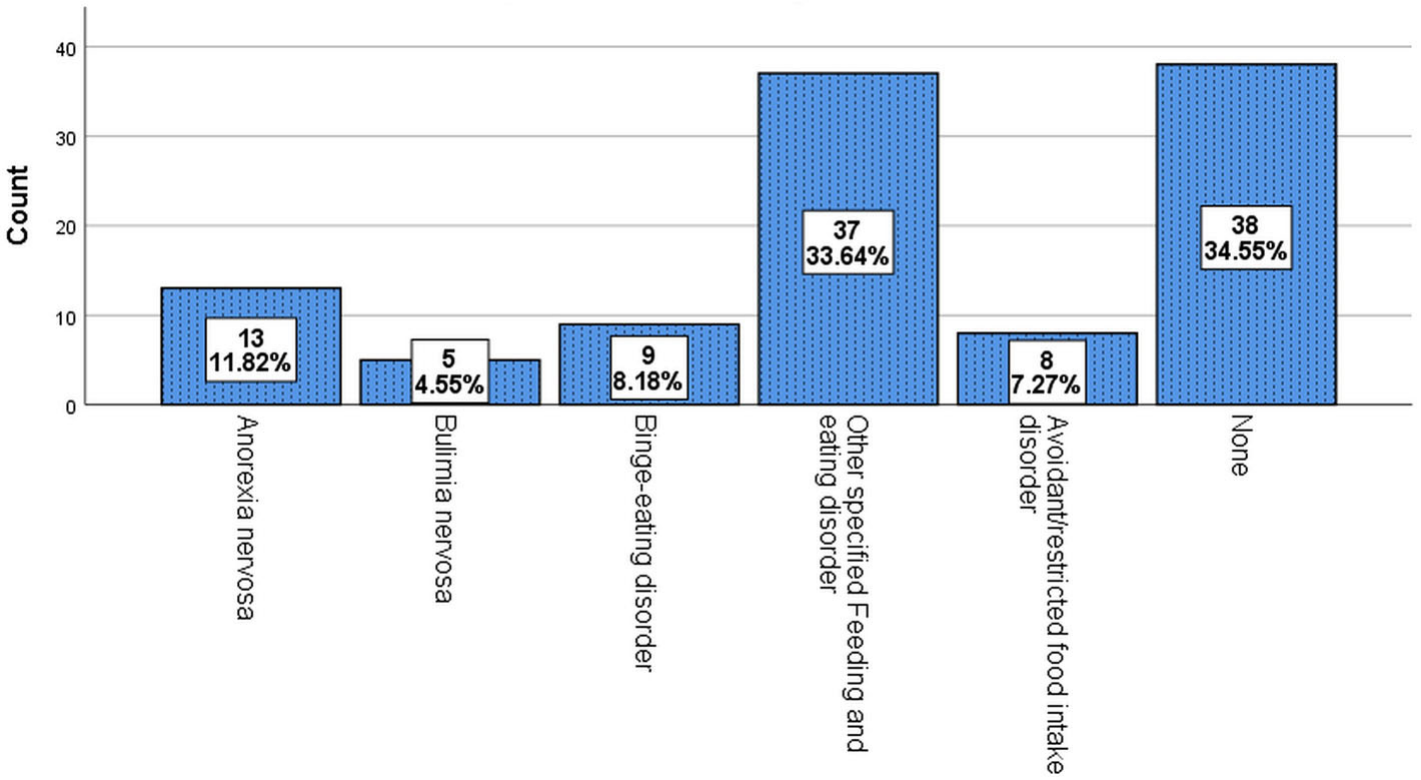
The prevalence of different types of FEDs in BPD + FEDs group (*n* = 72). *Note.* BPD: borderline personality disorder; FEDs: Feeding and eating disorders

**Fig. 2:**
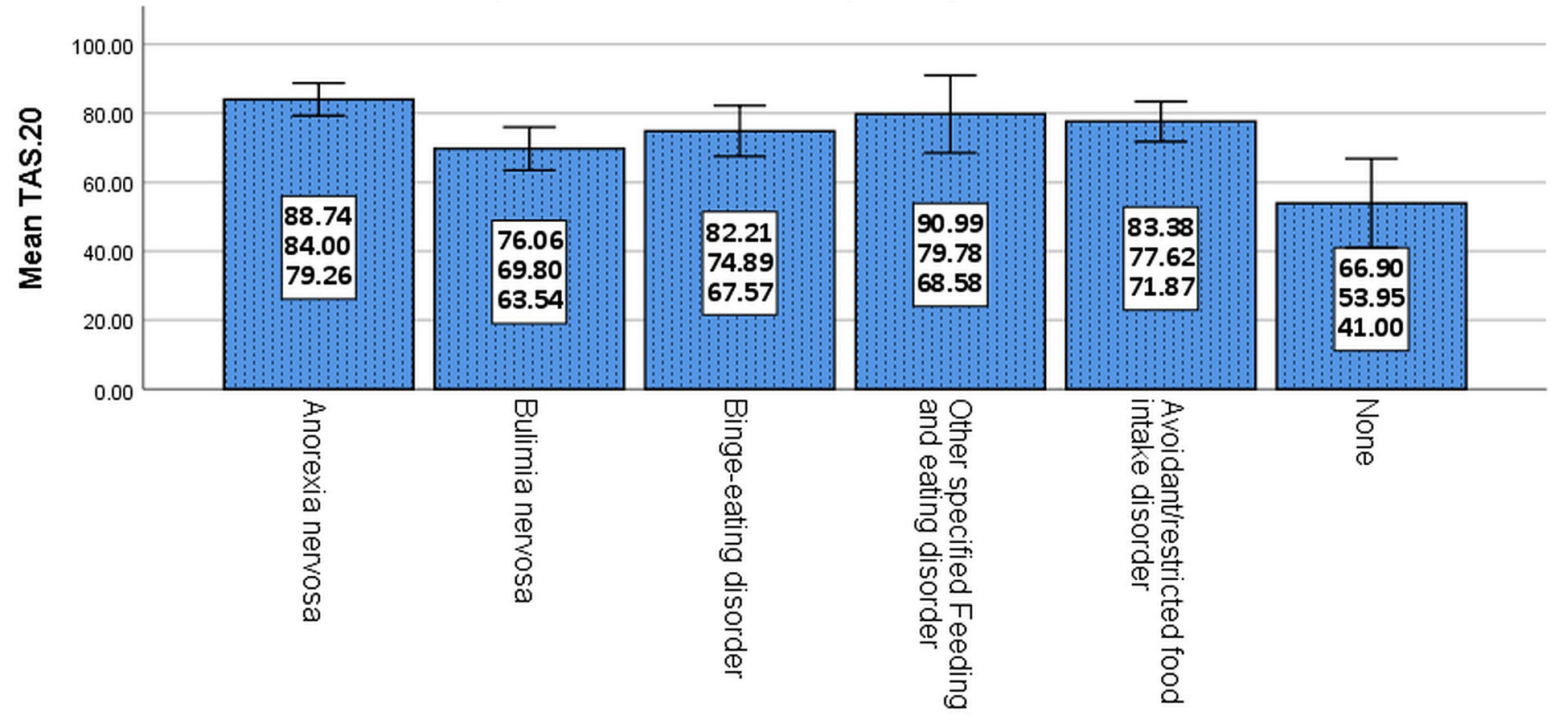
Total alexithymia scores (TAS) by different types of FEDs (Error bars: 95% CI, ± 1 SD) (n = 110). *Note.* ANOVA: F (5, 104) = 43.65, *p* < 0.001; Scheffé post-hoc test: Anorexia nervosa, Bulimia nervosa, Binge-eating disorder, Other specified feeding and eating disorders, Avoidant/restricted food intake disorder > None

The results of the SCID-5-RV interview indicated a 65.4% current prevalence of FEDs in patients with BPD. The highest and lowest frequencies were reported for other specified feeding and eating disorders (51.3%) and bulimia nervosa (6.9%), respectively (Fig. 1). No FEDs case was observed in the BPD only group through clinical interviewing. Although the mean scores of TAS-20 in the BPD + FEDs group were significantly higher than those of the BPD only group, this difference was not significant in terms of different types of FEDs (Fig. 2).

### Associations of study variables

The results of correlation in both the BPD only and BPD + FEDs groups suggested a significant positive correlation between the scores of EAT-26 and BAI (r = 0.58; *p* < 0.01), BDI-II (r = 0.52; *p* < 0.01), and TAS-20 (r = 0.59; *p* < 0.01). Furthermore, there was a significant positive correlation between the scores of TAS-20 and BAI (r = 0.67; *p* < 0.01) and BDI-II (r = 0.52; *p* < 0.01) (see Table 2).

**Table 2:** Correlations between BAI, BDI-II, TAS-20, and EAT-26 in BPD + FEDs group (*n* = 72)

Variables	M (SD)	1	2	3	4
1. BAI	58.69 (7.59)	1			
2. BDI-II	34.76 (8.35)	0.53^**^	1		
3. TAS-20	71.06 (15.31)	0.67^**^	0.52^**^	1	
4. EAT-26	22.71 (12.03)	0.58^**^	0.52^**^	0.59^**^	1

***Note.****BAI* Beck Anxiety Inventory, *BDI-II* Beck Depression Inventory-II, *EAT-26* 26-item Eating Attitudes Test, *TAS-20* 20-item Toronto Alexithymia Scale

^*^p < 0.05; ^**^p < 0.01; ^***^p < 0.001

### Mediation analysis

**Fig. 3:**
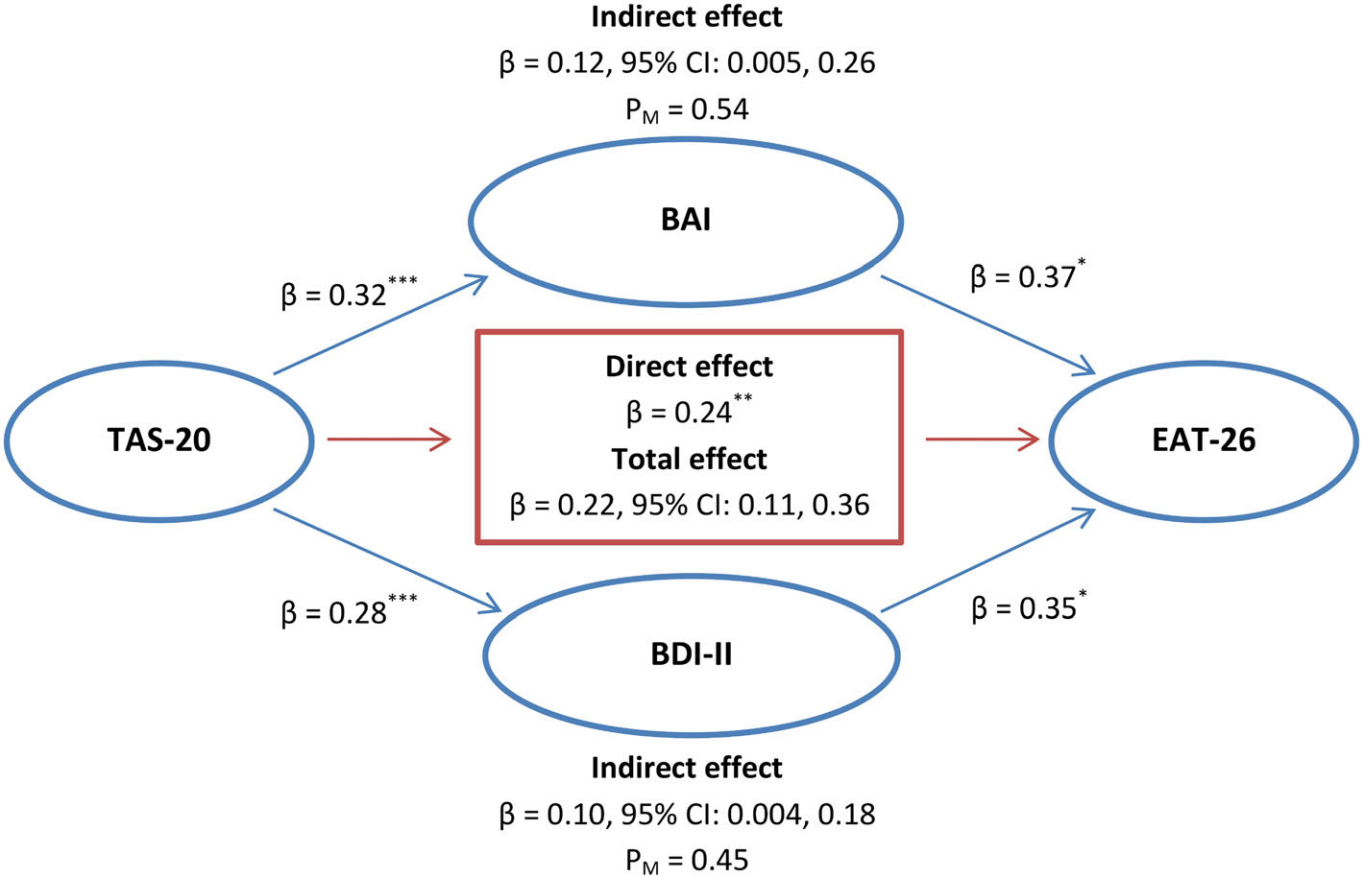
Illustration of the results of the mediation analysis described in the text, which tested BAI and BDI-II total scores (the measures of anxiety and depression, respectively) as the potential mediators of the relationship between TAS-20 and EAT-26 total scores (the measures of alexithymia and disordered eating behaviors, respectively) by controlling for sociodemographic variables (included age, gender, marital status, degree level, and income) in BPD + FEDs group (n = 72). *Note.* P_M_: Effect size (ratio of indirect to total effect) ^*^p < 0.05; ^**^p < 0.01; ^***^p < 0.001.

The mediation analysis was performed using Hayes’ PROCESS tool in SPSS (Model = 4, Bootstrap Samples = 5000). As hypothesized, a significant indirect effect of Alexithymia was direst observed on eating-disordered behaviors through anxiety and depression (β = 0.12, 95% CI: 0.005, 0.26; β = 0.10, 95% CI: 0.004, 0.18, respectively). The mediators (i.e., anxiety and depression) could account for roughly 54% (P_M_ = 0.54) and 45% (P_M_ = 0.45) of the total effect of alexithymia on EAT-26 total score, respectively. Hence, the overall hypothesis that anxiety and depression mediate the effect of alexithymia on eating-disordered behaviors was supported (Fig. 3).

## Discussion

In this study, there was a significant difference in the scores of BAI, BDI-II, TAS-20, EAT-26, and GHQ-28 among the three study groups. The highest scores were reported for the BPD + FEDs group, and the lowest scores were obtained for healthy controls. These findings were consistent with the results of the study performed by Eizaguirre et al. [16], Nowakowski et al. [12], and Gilboa-Schechtman et al. [18] who showed that the scores of TAS-20, BAI, and BDI-II in the BPD + FEDs group are higher than those of healthy controls. These results indicated that the BPD + FEDs patients had more difficulties in identifying and describing their emotions than BPD only patients and healthy controls potentially due to a greater degree of distress and less effective coping styles [12, 16].

Additionally, in this study, a higher FEDs prevalence was reported among patients with BPD compared to previous studies (65.4% vs. 14–53%). The highest and the lowest frequencies were reported for other specified feeding and eating disorders (51.3%) and bulimia nervosa (6.9%), respectively. In line with the findings, in the study performed by Zanarini et al. [2, 38], eating disorders not otherwise specified (i.e., diagnosis of other specified feeding and eating disorders in DSM-5) were the most common FEDs among patients with BPD. Moreover, the low rate of bulimia nervosa in patients with BPD has been contrary to much prior research [2–4, 38]. This is because FEDs frequency among patients with BPD has been investigated based on DSM-III-R and DSM-IV-TR criteria in previous studies, which are different from DSM-5 criteria. In addition, avoidant/restricted food intake disorder is a new diagnosis in DSM-5 that is very similar to anorexia nervosa, aside from the fact that there is no distress about body shape/size or fear of fatness in avoidant/restricted food intake disorder [28]. No subcategory of FEDs was observed in the only BPD group trough interviewing, suggesting that EAT-26 can be assumed as a useful screening instrument (with a cutoff point of above 20) [25] for diagnosing different types of FEDs in patients with BPD.

Although the highest mean score of TAS-20 in this study was related to anorexia nervosa, there was no significant difference between the scores of different types of FEDs. This finding was consistent with the results of the studies performed by Cochrane et al. [9], Troop et al. [39], and Berthoz et al. [40]. However, it was inconsistent with the studies performed by Eizaguirre et al. [16], Nowakowski et al. [12], and Gilboa-Schechtman et al. [18], who showed that mean score of TAS-20 in patients with anorexia nervosa was significantly higher than that in other types of FEDs. However, the findings may reflect insufficient power as there were not many anorexia nervosa cases in the study sample. Thus, it may represent a Type 2 error rather than a sign that anorexia nervosa does not present with high alexithymia.

Together the results demonstrated a considerable positive correlation between the scores of EAT-26 and BAI, BDI-II, and TAS-20. Moreover, there was a significant positive correlation between the scores of TAS-20 and BAI and BDI-II. These findings suggested that, in patients affected by BPD + FEDs, alexithymia was closely related to anxiety and depression. Here, this question is raised: “Is alexithymia either a state variable or a trait variable?” There is strong evidence that alexithymia is not only a by-product of FEDs symptomatology. However, there is mixed evidence of whether alexithymia is independent of general distress [12, 16, 18]. Nevertheless, several studies have indicated that elevated alexithymia scores become eliminated by controlling the effect of distress. However, some other studies have suggested that, in patients with FEDs, elevated alexithymia scores remain constant even by controlling the effect of anxiety and depression [12]. In this study, the mediation analysis showed that the role of alexithymia in the prediction of eating-disordered behaviors remained unchanged even by controlling the effect of anxiety and depression. Accordingly, alexithymia may be a trait rather than a state. In this regard, Schmidt et al. [41] stated that pharmacotherapy with antidepressants can only decrease depression level in patients with high levels of alexithymia and depression. Moreover, genetic studies have concluded that alexithymia has its own heritability component that cannot be fully explained by depression or genetic susceptibility to general distress and psychopathology [42]. As a result, although the relevant evidence is mixed, it is recommended that clinical attention should be paid to alexithymia as a distress-independent construct [12]. However, the cross-sectional design of this study prevents an understanding of relationships’ exact nature, particularly with respect to causality.

The findings have many implications for nosology and interventions. First, the FEDs among patients with BPD are likely to be a subtype of emotional disorders that, in turn, should affect the prediction of the course of these afflictions. For example, if depressive episodes as a primary disorder constitute the core of these conditions, the risk of developing depressive episodes remains high, even with the recovery from the FEDs. Second, this nosological shift may also affect the type of treatment. If anxiety and depression, for instance, are diagnosed as primary or secondary disorders, the treatment will be different. Third, emotional awareness normalization and emotion regulation may be regarded as recovery markers beyond symptom reduction [18].

However, there were some limitations faced in this study. First, the cross-sectional design avoided the precise understanding of relationships’ nature, especially concerning causality. It is essential to differentiate between primary and secondary alexithymia since cognitive therapies are more effective in primary alexithymia than dynamic ones [43]. However, secondary alexithymia responds to a wide range of treatments. Second, due to the small sample size (particularly among different types of FEDs in BPD + FEDs group) and participant selection from a narrow age-range and specific geographical region, the study results should be generalized to other societies with caution. Third, the adults may report different levels of alexithymia in response to certain life events based on the individual difference model. Thus, future studies should focus on some methodological constraints, including sole reliance on self-report measures of alexithymia (due to memory bias and demand characteristics), lack of longitudinal and experimental studies, lack of ethnic differences, limited age range, and negative emotions exclusively.

## Conclusions

In conclusion, this study showed that BPD + FEDs patients found it difficult to identify and describe emotions. However, there was no significant difference in the level of alexithymia among different types of FEDs. The results also suggested that other specified feeding and eating disorders may be the most prevalent FEDs in patients with BPD. Besides, anxiety and depression could mediate the relationship between alexithymia and eating-disordered behaviors in patients with BPD. Accordingly, the FEDs treatment protocols among patients with BPD need to concentrate on emotions and emotion regulation to assess alexithymia, anxiety, and depression conveniently, and determine whether alexithymia precedes FEDs. Nonetheless, further longitudinal and experimental studies with a larger variety of measures to explore psychopathology could contribute to clarifying all these questions and ascertaining primary and secondary alexithymia in FEDs.

## Data Availability

The datasets generated and analyzed during the current study are not publicly available because no consent was obtained from the participants. However, the data are available by the corresponding author on a reasonable request.

## Abbreviations

ANOVA: Analysis of variance
BAI: Beck Anxiety Inventory
BDI-II: Beck Depression Inventory-II
BPD: Borderline personality disorder
BPI: Borderline Personality Inventory
EAT-26: 26-item Eating Attitudes Test
FEDs: Feeding and eating disorders
GHQ-28: 28-item General Health Questionnaire
SCID-5-RV: Structured Clinical Interviews for DSM-5: Research Version
SCID-5-PD: Structured Clinical Interview for DSM-5 Personality Disorders
TAS-20: 20-item Toronto Alexithymia Scale
